# Antibiogram and recent incidence of multi-drug resistant carbapenemase producing Escherichia coli isolated from paediatric patients

**DOI:** 10.12669/pjms.36.2.928

**Published:** 2020

**Authors:** Sumbal Nosheen, Nadeem Irfan Bukhari, Hasan Ejaz, Nasir Abbas

**Affiliations:** 1 Dr. Sumbal Nosheen, M.Phil. Punjab University College of Pharmacy (PUCP), University of the Punjab, Lahore, Pakistan; 2 Dr. Nadeem Irfan Bukhari, Ph.D. Punjab University College of Pharmacy (PUCP), University of the Punjab, Lahore, Pakistan; 3 Dr. Hasan Ejaz, Ph.D. Department of Clinical Laboratory Sciences, College of Applied Medical Sciences, Jouf University, Sakaka, Saudi Arabia; 4 Dr. Nasir Abbas, Ph.D. Punjab University College of Pharmacy (PUCP), University of the Punjab, Lahore, Pakistan

**Keywords:** Antibiotic resistance, Multi-drug resistance, Escherichia coli, Carbapenemase producers

## Abstract

**Objective:**

To gauge the recent breadth of MDR E. coli along with antibiogram of carbapenemase producing (CP) E. coli among children from an institute which receives patients from all over Punjab.

**Methods:**

The bacterial strains of E. coli isolated from various specimens of patients were collected from April 2017 to August 2018 and processed using standard biochemical tests and API 20E system (bioMerieux). Phenotypic screening for CP E. coli was done by the modified Hodge test, whereas antibiotic susceptibility testing was done with Kirby-Bauer disc diffusion technique.

**Results:**

Total of 6,468 bacterial strains were isolated, out of which 1,552 (24%) were E. coli. Carbapenem resistance was observed in 245 (16%) strains, amongst which 113 (46%) were confirmed to be CP. E. coli isolated from males were higher as compared to females (p<0.05). Majority of the organisms were isolated from blood (37.2%) samples. The hospital discharged about 65% of patients, while 23% left against medical advice. Overall MDR amongst E. coli was 93.26%. Colistin sulphate (15.9%) and nitrofurantoin (16.8%) showed the most efficacy followed by amikacin (15%) and fosfomycin (10.6%).

**Conclusion:**

The isolation of high number of MDR E. coli amongst the paediatric patients is worrisome, which could serve as a potential source of horizontal genes transfer to other genera.

## INTRODUCTION

Multiple-drug resistance (MDR) is developed in microorganisms against two or more antibiotics concomitantly due to either production of bacterial enzymes against them, modification in bacterial penicillin-binding proteins, their extrusion by efflux pump or mutations in transferable genes to other bacteria through mobile genetic elements.^1^

Most Gram-negative microbes like *E. coli* are resistant to various antibiotics classes including penicillins, cephalosporin, macrolides and aminoglycosides. Such MDR infections are difficult to treat in compromised paediatric patients which may lead to severe consequences like delayed treatment, prolonged hospitalization, complications, repeated hospital visits and even deaths if could not be managed.^2^

Carbapenems have a strong affinity for the penicillin-binding proteins present on bacterial membrane and are stable against extended-spectrum beta-lactamase and therefore shows high permeability in the bacterial outer membrane.^3^ As the bacteria producing carbapenemases have begun to emerge, MDR infections are becoming more formidable challenge.^4^

Carbapenem resistant *E. coli* has now considered among most important nosocomial pathogens that can cause a variety of infections including urinary tract infections (UTI), intestinal and extra-intestinal ailments like diarrhoea and neonatal meningitis.^5^ The carbapenems are regarded as an empirical treatment choice for all severe infections due to AmpC and extended-spectrum β-lactamase producing *E. coli*. An increase in the prevalence of the above diseases has been documented in recent years with wide variations in incidence among different regions of the world.^6^

In the US, *E. coli* induced UTIs among paediatric patients to result in approximately eight million health practitioner visits per year.^7^ Therapeutic options for the treatment of such bacteria are now limited, and the delays in the detection of carbapenemase production by bacteria mostly leads to the treatment failure, prolonged hospital stay, increased morbidity, mortality and healthcare cost.^8^ Thus, evaluation of the antimicrobial resistance pattern of carbapenemase producing *E. coli* in local paediatric patients has been warranted. We aimed the study to evaluate the incidence of multi-drug resistant carbapenemase producing *E. coli* in paediatric patients attending a tertiary care hospital of Lahore, Pakistan. Likewise, the patient’s statistic information is a valuable supplement to sensitivity data in distinguishing patient groups at high danger of infection.

## METHODS

This prospective study was carried out on *E. coli* from all of the pathological samples received at the Department of Microbiology from admitted and ambulatory patients attending hospital, received at the Department of Microbiology, The Children’s Hospital Lahore from April 2017 to August 2018 after approval from the institutional ethical committee. We processed a total number of 52,163 specimens for 17 months for the isolation of carbapenemase producing *E. coli*.

The specimens of blood, ear swab tip, central venous pressure (CVP) tip, peritoneal dialysis (PD) catheter, pus, urine, tracheal secretion, cerebrospinal fluid (CSF), endotracheal tube (ETT), and wound swab were cultured on the Blood and MacConkey agar plates. After incubation, the strains were identified morphologically and by using Gram’s stain, various biochemical tests and API 20 E.^9^ Phenotyping for carbapenemase detection in *E. coli* in the specimen was done by the modified Hodge test (MHT) and combined disc test (CDT). The appearance of clover leaf-like indentation showed MHT positive carbapenemase producing *E. coli*.^10^ The non-repetitive isolates, resistant to any of the carbapenem drug were included from the study. The demographic data of the enrolled cases was obtained from the patient files.

The data were analyzed by using SPSS version 23.0. Gender was compared for output with different wards, specimens and outcomes like mortality and discharge by using the Chi-square test. A p-value <0.05 was considered significant. Friedman’s test was employed to compare the difference in the effect of antibiotics on the strains of carbapenemase producing *E. coli*.

## RESULTS

We isolated a total of 6,468 (12.3%) different bacterial isolates out of which 1,552 (24%) were identified as *E. coli*. Resistance against carbapenems was observed in 245/1552 (16%) isolates, amongst which 113/245 (46%) were found to be CP by MHT and CDT.

The highest number of isolated bacterial strains was 42 (37.2%) from urine and 40 (35.4%) from the blood. There were 22 (19.5%) samples collected from the neonatal unit, followed by 15 (13.3%) from oncology and 14 (12.4%) from ICUs. A total of 56 (49.6%) CP strains were recovered from the females while 57 (50.4%) from male patients. The overall outcome showed that 73 (65%) patients were discharged after successful treatment while 23 (20%) patients left against the medical advice (LAMA). There were 17 (15%) cases of mortality. No statistical association (p>0.05) was found between the gender with the outcome, specimen and wards (Table-I).

**Table-I:** Demographic data of patients infected with CP *E. coli* infections (n=113).

Specimen	Frequency (n)	Percentage (%)
Blood	42	37.20%
Ear Swab Tip	3	2.70%
CVP Tip	4	3.50%
PD Catheter	4	3.50%
Pus	6	5.30%
Urine	40	35.40%
Tracheal secretion	2	1.80%
CSF	9	8%
ETT	1	0.90%
Wound Swab	2	1.80%

*Wards*	*Frequency (n)*	*Percentage (%)*

Neonatal Unit	22.0	19.5%
Development Ward	2.0	1.8%
Surgery Ward	4.0	3.5%
All Intensive Care Units (ICUs)	14.0	12.4%
Medical Emergency	2.0	1.8%
Haematology/Oncology Unit	15.0	13.3%
Cardiology Ward	1.0	0.9%
Outdoor Patients (OPD)	7.0	6.2%
Urology Ward	3.0	2.7%
General Medical Unit-I	3.0	2.7%
General Medical Unit-II	11.0	9.7%
General Medical Unit-III	4.0	3.5%
General Medical Unit-IV	3.0	2.7%
Nephrology Ward	15.0	13.3%
Gastroenterology Ward	3.0	2.7%
Neurology ward	1.0	0.9%
Neuro-Surgery ward	3.0	2.7%

*Gender*	*Frequency (n)*	*Percentage (%)*

Female	56	49.6%
Male	57	50.4%

*Outcome*	*Frequency (n)*	*Percentage (%)*

Discharge	73	65%
Death	17	15%
LAMA (left against medical advice)	23	20%

Antibiogram was determined by the Kirby Bauer disc diffusion method. The antibiotics used were those as given in Table-II. The diameter of each zone of inhibition was identified and interpreted as sensitive, intermediate or resistant as described in CLSI guidelines.^10^ E-test was also used to measure the minimum inhibitory concentration of anti-microbial susceptibility of *E. coli* against colistin. The intersection point of the drop-shaped inhibition zone and the graded E-strip showing the inhibitory concentration of colistin was interpreted according to CLSI. All strains showing lower susceptibility to carbapenems like imipenem and meropenem were screened to be CP by using MHT and CDT.^10^

**Table-II:** Antibiogram of carbapenemase producing *E. coli* (n=113).

Antibiotics*	Resistant		Sensitive		Intermediate	

	No.	%	No.	%	No.	%
***Cephalosporin***						
Cefoxitin	95	84.1	12	10.6	6	5.3
Cefuroxime	109	96.5	4	3.5	0	0.0
Cefixime	110	97.3	3	2.7	0	0.0
Ceftazidime	113	100.0	0	0.0	0	0.0
Ceftriaxone	111	98.2	2	1.8	0	0.0
Cefotaxime	113	100.0	0	0.0	0	0.0
Cefepime	104	92.0	5	4.4	4	3.5
***Aminoglycosides***						
Amikacin	96	85.0	17	15.0	0	0.0
Gentamycin	108	95.6	5	4.4	0	0.0
Tobramycin	110	97.3	3	2.7	0	0.0
***Polypeptide Polymyxin Antibiotics***						
Colistin	89	78.8	18	15.9	6	5.3
***Quinolones***						
Nalidixic acid	108	95.6	5	4.4	0	0.0
***Fluoroquinolones***						
Levofloxacin	108	95.6	5	4.4	0	0.0
Moxifloxacin	112	99.1	0	0.0	1	0.9
Ciprofloxacin	106	93.8	5	4.4	2	1.8
Norfloxacin	102	90.3	11	9.7	0	0.0
***Monobactams***						
Aztreonam	107	94.7	6	5.3	0	0.0
***Nitrofurans***						
Nitrofurantoin	92	81.4	19	16.8	2	1.8
***Carbapenems***						
Imipenem	105	92.9	4	3.5	4	3.5
Meropenem	103	91.2	5	4.4	5	4.4
***Others***						
Fosfomycin	101	89.4	12	10.6	0	0.0
Pipemidic acid	112	99.1	1	0.9	0	0.0
***Combination Antibiotics***						
Co-amoxiclav	109	96.5	4	3.5	0	0.0
Piperacillin-tazobactam	105	92.9	2	1.8	6	5.3
Co-trimoxazole	110	97.3	3	2.7	0	0.0
Cefoperazone-sulbactam	102	90.3	4	3.5	7	6.2

* p<0.05 using Friedman test used to see the difference in the effects of antibiotics

In antimicrobial susceptibility testing, colistin sulphate (15.9%) and nitrofurantoin (16.8%) were found to be the most effective antibiotic in vitro followed by amikacin (15%) and fosfomycin (10.6%). The overall MDR among *E. coli* strains was found to be 93.26%. The bacterial isolates were found to be 100% resistant to cefotaxime and ceftazidime. Lower susceptibility rates were seen for cefoperazone-sulbactam (3.5%) and piperacillin-tazobactam (1.8%). The p-value <0.05 indicated that there was a difference in the effects of antibiotics (Table-II).

## DISCUSSION

In this study, we included the patients received at a prominent paediatric hospital at Lahore, which is serving such patient population from all over the Punjab province and reported 16% CP *E. coli*. A study at Indian hospital identified 22 *E. coli* isolates, all of which showed complete resistance against carbapenem antibiotics.^11^ In another study in Los Angeles, USA, eleven *E. coli* strains were identified out of which 5 (45.4%) were CP.^12^ A study conducted at Karachi hospital reported the maximum cases of CP from urine samples (40%).^13^ However, in our study, the maximum of CP *E. coli* were isolated from blood samples (37.2%).

In our study, only 6.2% CP *E. coli* strains were isolated from outpatients while 93.8% from hospitalized patients which is in contrast to an Iranian study, where a lower frequency of *E. coli* (33%) was isolated from inpatients than from outpatients (66%).^14^ A study including seven US hospitals showed that 51% of MDR CP *E. coli* were isolated from ICUs followed by medical ward (38%) and surgery unit (20%).^7^ These remarkably high prevalence values are unprecedented with the frequency of our study in which only 12.4% cases were from ICUs, 18.6% from general medical and surgery ward. The reason for the higher risk of nosocomial infections in ICUs patients may be due to the critical condition and suppressed immunity of the patients needing intensive care.

The frequency of CP *E. coli* strains was higher in males 57 (50.4%) than females 56 (49.6%) in this study. Contrarily, *E. coli* infection was found to be higher in females (71%) than in male patients (55%) in another study.^15^ The reason for such a contradiction with our result may be due to the fact that the parents in the developing countries bring male children more to the hospital in comparison to the female.

In a study conducted at St. Louis teaching hospital, the mortality rate of patients with MDR *E. coli* infection was 61.9%which is quite high in comparison to our study (15%).^16^ Mortality related to such infections can be predetermined to be present with patients acquiring nosocomial infections during the hospital stay or due to interventions like surgeries.

The present study showed an alarming increased rate in MDR in *E. coli* (93.26%) as compared to other study carried out in Sudan (57%).^17^ A US study reported 92% *E. coli* to be sensitive to colistin, which is higher than our study (15.9%).^18^ The current study expressed that most of *E. coli* were concurrently MDR against different antibiotic classes like cephalosporins, aminoglycosides, fluoroquinolones and carbapenems. Similar reports by others showed higher resistance to these antibiotics.^14^ A possible reason for the high co-resistance found in such strains could be the modification of bacterial genes; hence producing enzymes like metallo-beta-lactamases, resulting in increased resistance. A study from India also reported high resistance to CP strains to aminoglycosides, beta-lactams, sulphonamides and fluoroquinolones.^11^ Various factors like polypharmacy, lesser trends of using targeted antibiotics and not promoting bacterial identification tests contribute towards higher resistance patterns of MDR *E. coli*.

Sahm *et al*. reported 7.1% MDR *E. coli* in 2000.^19^ A very high incidence of metallo-beta-lactamase (97.8%) producing bacteria resistant to carbapenems, aminoglycosides, cephalosporin and fluoroquinolones have been reported in paediatric patients.^20^ The relevance of genetic background and the MDR bacteria with a high level of resistance to carbapenems, co-amoxiclav, ampicillin, aztreonam and cephalosporins have been reported amongst the paediatric patients of Pakistan.^21^ Pharmacists can play an integral role in depressing the rapid spread of resistance in pathogens among paediatric patients by involving themselves at clinical levels and promoting rational use of drugs, decreasing the use of un-targeted potent antibiotics as empirical therapy as well as polypharmacy and facilitating the formulation of future antibiotic policies. The study was conducted in the largest tertiary care hospital of Punjab; nevertheless, it was not possible to obtain data on regional and locality basis, a limitation of this study.

## CONCLUSION

This study demonstrated high number of CP producing *E. coli* from clinical samples of admitted and outpatients from a tertiary care paediatric hospital. The pathogens were combatively less resistant to colistin and amikacin, but some other resistance mechanism could lead to their reduced sensitivity. This high rate of resistance could be a direct result of increased trends towards the use of untargeted potent antibiotics as well as that of antibiotic polypharmacy in hospitals as empirical therapy. The understanding of mechanisms of horizontal transfer of genes can be helpful in the development of new policies to fight this health issue of MDR infections.

### Authors’ Contribution:

**SN:** Translated idea into experimental, designed the study and written the manuscript, is responsible for integrity of research.

**HE:** Jointly conceived idea and supervised the work with NIB, contributed in the specimen collection and editing of the manuscript.

**NA:** Helped in study design, helped results interpretation and editorial help for manuscript.

**NIB:** Conceived the idea with HE, supervised the work, performed statistical analysis and finally approved the manuscript.

## References

[1] Hollis A, Ahmed Z (2013). Preserving antibiotics, rationally. N Engl J Med.

[2] Lim K-T, Yasin R, Yeo C-C, Puthucheary S, Thong K-L (2009). Characterization of multidrug resistant ESBL-producing Escherichia coli isolates from hospitals in Malaysia. BioMed Res Int.

[3] Nordmann P, Gniadkowski M, Giske C, Poirel L, Woodford N, Miriagou V (2012). Identification and screening of carbapenemase producing Enterobacteriaceae. Clin Microbiol Infect.

[4] Girlich D, Poirel L, Nordmann P (2011). Value of the modified Hodge test for detection of emerging carbapenemases in Enterobacteriaceae. J Clin Microbiol.

[5] Bergeron CR, Prussing C, Boerlin P, Daignault D, Dutil L, Reid-Smith RJ (2012). Chicken as reservoir for extraintestinal pathogenic Escherichia coli in humans, Canada. Emerg Infect Dis.

[6] Rolain J, Parola P, Cornaglia G New Delhi metallo beta lactamase (NDM-1):Towards a new pandemia?Clin Microbiol Infect. 2010;.

[7] Wisplinghoff H, Bischoff T, Tallent SM, Seifert H, Wenzel RP, Edmond MB (2004). Nosocomial bloodstream infections in US hospitals:Analysis of 24,179 cases from a prospective nationwide surveillance study. Clin Infec Dis.

[8] Mehrgan H, Rahbar M, Arab-Halvaii Z (2009). High prevalence of extended-spectrum beta-lactamase-producing Klebsiella pneumoniae in a tertiary care hospital in Tehran, Iran. J Infect Dev Ctries.

[9] Cheesbrough M (2006). District Laboratory Practice in Tropical Countries:Cambridge University Press.

[10] Clinical and Laboratory Standards Institute (CLSI) (2017). Performance Standards for Antimicrobial Susceptibility Testing.

[11] Muir A, Weinbren M (2010). New Delhi metallo-β-lactamase:A cautionary tale. J Hosp Infect.

[12] Pannaraj PS, Bard JD, Cerini C, Weissman SJ (2015). Paediatric carbapenem-resistant Enterobacteriaceae in Los Angeles, California, a high-prevalence region in the United States. Pediatr Infect Dis J.

[13] Khan FZ, Nawaz T, Mirani ZA, Khan S, Raza Y, Kazmi SU (2018). Study of class 1 integrons in multidrug-resistant uropathogenic Escherichia coli isolated from different hospitals in Karachi. Iran J Med Sci.

[14] Fazeli H, Moghim S, Zare D (2018). Antimicrobial Resistance Pattern and Spectrum of Multiple-drug-resistant Enterobacteriaceae in Iranian Hospitalized Patients with Cancer. Adv Biomed Res.

[15] Magliano E, Grazioli V, Deflorio L, Leuci AI, Mattina R, Romano P (2012). Gender and age-dependent etiology of community-acquired urinary tract infections. Sci World J.

[16] Ibrahim EH, Sherman G, Ward S, Fraser VJ, Kollef MH (2000). The influence of inadequate antimicrobial treatment of bloodstream infections on patient outcomes in the ICU setting. Chest.

[17] Ibrahim ME, Bilal NE, Hamid ME (2012). Increased multi-drug resistant Escherichia coli from hospitals in Khartoum state, Sudan. Afr J Health Sci.

[18] Deshpande LM, Jones RN, Fritsche TR, Sader HS (2006). Occurrence and characterization of carbapenemase-producing Enterobacteriaceae:Report from the SENTRY Antimicrobial Surveillance Program (2000-2004). Microb Drug Resist.

[19] Sahm DF, Thornsberry C, Mayfield DC, Jones ME, Karlowsky JA (2001). Multidrug-Resistant Urinary Tract Isolates of Escherichia coli:Prevalence and Patient Demographics in the United States in 2000. Antimicrob. Agents Chemother.

[20] Javed H, Ejaz H, Zafar A, Rathore AW, ul Haq I (2016). Metallo-beta-lactamase producing Escherichia coli and Klebsiella pneumoniae:A rising threat for hospitalized children. J Pak Med Assoc.

[21] Heinz E, Ejaz H, Scott JB, Wang N, Gujaran S, Pickard D (2019). Resistance mechanisms and population structure of highly drug resistant Klebsiella in Pakistan during the introduction of the carbapenemase NDM-1. Sci Rep.

